# Supplementary material for: Association of Abnormal Iron Status with the Occurrence and Prognosis of Peritoneal Dialysis-Related Peritonitis: A Longitudinal Data-Based 10-Year Retrospective Study
Source: Nutrients. 2022 Apr 13;14(8):1613. doi: 10.3390/nu14081613 (PMC9027868; doi:10.3390/nu14081613)

Table S1. Univariate and multivariate negative binomial regression analysis for baseline iron status and the occurrence of peritonitis

	Univariate model		Multivariate model#	
	OR (95% CI)	*P* value	AOR (95% CI)	*P* value
Age	1.02 (1.01–1.03)	**<0.001**	1.02 (1.00–1.03)	**0.01**
Male gender	0.83 (0.64–1.07)	0.15	0.92 (0.71–1.20)	0.53
Diabetes	0.81 (0.59–1.11)	0.19	-	-
CCI	1.11 (1.04–1.20)	**0.002**	0.94 (0.84–1.06)	0.30
ALB	0.94 (0.91–0.96)	**<0.001**	0.94 (0.92–0.97)	**<0.001**
Hb	0.99 (0.99–1.01)	0.26	-	-
Hs-CRP	0.99 (0.98–1.02)	0.96	-	-
N/L ratio	1.07 (0.99–1.15)	0.11	-	-
Iron status				
RIS	Ref.		Ref.	
AID	1.30 (0.93–1.81)	0.13	1.27 (0.91–1.77)	0.17
FID	1.08 (0.75–1.55)	0.68	1.01 (0.71–1.44)	0.96
HIS	1.12 (0.77–1.63)	0.56	1.14 (0.79–1.65)	0.50

OR, odd ratio; CI, confidence interval; ALB: albumin; CCI: Charlson's comorbidity index; Hs-CRP: high-sensitivity C-reactive protein; N/L ratio: neutrophil/lymphocyte ratio; RIS: reference iron status; AID: absolute iron deficiency; FID: functional iron deficiency; HIS: high iron status

Table S2. Univariate and multivariate cox regression analysis for baseline iron status and first episode of peritonitis

	Univariate model		Multivariate model	
	HR (95% CI)	*P* value	AHR (95% CI)	*P* value
Age	1.02 (1.01–1.03)	**<0.001**	1.01 (0.99–1.03)	0.06
Male gender	0.99 (0.78–1.27)	0.96	1.03 (0.79–1.33)	0.84
Diabetes	1.31 (0.99–1.73)	0.06		
CCI	1.15 (1.08–1.23)	**<0.001**	1.02 (0.91–1.13)	0.77
ALB	0.94 (0.91–0.96)	**<0.001**	0.95 (0.93–0.98)	**<0.001**
Hb	0.99 (0.99–1.01)	0.14	-	-
Hs-CRP	1.01 (0.99–1.02)	0.22	-	-
N/L ratio	1.15 (1.08–1.23)	**<0.001**	1.12 (1.05–1.20)	**0.001**
Iron status				
RIS	Ref.		Ref.	
AID	1.05 (0.76–1.43)	0.79	1.05 (0.75–1.45)	0.79
FID	1.15 (0.83–1.61)	0.40	1.04 (0.74–1.45)	0.84
HIS	1.20 (0.83–1.73)	0.33	1.22 (0.84–1.76)	0.30

HR, hazard ratio; CI, confidence interval; ALB: albumin; CCI: Charlson's comorbidity index; Hs-CRP: high-sensitivity C-reactive protein; N/L ratio: neutrophil/lymphocyte ratio; RIS: reference iron status; AID: absolute iron deficiency; FID: functional iron deficiency; HIS: high iron status

Table S3. Longitudinal data of Hb, iron biomarkers and CRP at each follow-up phase

Follow-up phase	Hb (g/dL), median (IQR)	Ferritin (μg/L), median (IQR)	Serum iron (μmol/L), median (IQR)	TSAT (%), median (IQR)	Hs-CRP (mg/L) , median (IQR)
T1	11.1 (9.90–12.2)	147 (63.7–282)	10.3 (6.80–14.0)	18.3 (12.2–27.5)	1.80 (0.67–5.55)
T2	11.3 (10.2–12.3)	170 (87.9–299)	12.5 (9.00–16.9)	23.5 (16.0–32.6)	1.69 (0.65–5.10)
T3	11.1 (9.85–12.1)	155 (86.3–291)	12.6 (8.60–16.6)	23.3 (15.4–33.0)	1.84 (0.67–6.02)
T4	11.0 (9.90–12.0)	155 (84.1–265)	12.1 (8.55–16.6)	22.5 (14.9–32.5)	1.90 (0.69–6.06)
T5	11.1 (9.90–12.1)	142 (73.7–258)	12.3 (8.40–16.0)	23.2 (15.5–33.2)	1.95 (0.67–5.55)
T6	11.0 (9.58–12.1)	139 (77.7–265)	12.1 (8.50–15.9)	23.9 (16.1–32.5)	1.96 (0.72–6.81)
T7	11.0 (9.60–12.0)	123 (66.7–223)	11.8 (8.50–15.7)	22.4 (16.6–32.3)	1.96 (0.73–6.32)
T8	10.9 (9.40–12.0)	118 (61.9–228)	11.4 (8.00–15.6)	22.7 (16.2–31.8)	2.46 (0.80–7.04)
T9	11.0 (9.20–12.1)	114 (59.8–222)	11.7 (8.29–16.4)	24.2 (16.5–34.5)	2.40 (0.76–6.49)
T10	10.6 (9.40–11.7)	115 (55.9–211)	11.7 (8.40–16.0)	24.1 (16.5–35.3)	2.52 (0.99–7.07)
T11	10.9 (9.70–11.9)	121 (57.2–231)	11.5 (7.77–15.4)	23.9 (16.6–33.4)	2.60 (0.89–7.17)
T12	11.0 (9.50–11.9)	102 (50.1–199)	12.2 (8.88–15.6)	26.6 (19.2–34.5)	2.42 (0.91–7.06)
T13	11.0 (9.68–12.1)	103 (59.2–222)	11.4 (8.55–14.9)	26.2 (18.1–34.2)	3.23 (1.47–8.03)
T14	10.9 (9.60–12.0)	107 (61.7–212)	12.1 (9.10–15.9)	26.4 (19.6–35.6)	2.92 (1.27–10.0)
T15	11.0 (9.60–12.3)	102 (53.5–235)	11.3 (8.40–15.2)	24.5 (18.3–33.0)	3.24 (0.91–8.72)
T16	10.9 (9.53–11.9)	108 (66.2–254)	12.0 (8.96–15.4)	25.8 (19.0–34.1)	3.19 (0.81–8.14)
T17	11.3 (10.0–11.9)	119 (63.2–226)	11.2 (8.40–14.1)	24.2 (17.3–32.3)	3.89 (0.99–9.50)

T18	11.8 (9.95–12.8)	108 (59.8–235)	12.2 (9.23–17.3)	27.3 (21.0–43.0)	2.34 (1.00–9.38)
T19	11.0 (9.05–12.2)	146 (66.5–262)	10.7 (8.60–15.6)	25.6 (19.1–36.2)	4.19 (0.84–9.65)
T20	11.4 (9.66–12.1)	141 (66.9–218)	12.3 (10.1–18.7)	26.9 (21.1–32.3)	1.01 (0.55–6.87)

Hb: hemoglobin; TSAT: transferrin saturation; Hs-CRP: high-sensitivity C-reactive protein

Table S4. The distribution of iron status records at each follow-up phase

Follow-up phase	Iron status				All (include NC), n (%)
	RIS, n (%)	AID, n (%)	FID, n (%)	HIS, n (%)	
T1	369 (24.9)	148 (10.0)	134 (9.0)	118 (8.0)	1483 (100)
T2	325 (27.8)	57 (4.9)	54 (4.6)	141 (12.0)	1171 (100)
T3	293 (27.8)	58 (5.5)	59 (5.6)	121 (11.5)	1053 (100)
T4	282 (30.3)	55 (5.9)	51 (5.5)	98 (10.5)	932 (100)
T5	243 (28.9)	39 (4.6)	44 (5.2)	90 (10.7)	842 (100)
T6	196 (26.9)	40 (5.5)	39 (5.3)	79 (10.8)	729 (100)
T7	168 (27.5)	36 (5.9)	24 (3.9)	42 (6.9)	610 (100)
T8	149 (28.2)	39 (7.4)	18 (3.4)	41 (7.8)	529 (100)
T9	123 (26.5)	35 (7.5)	13 (2.8)	34 (7.3)	465 (100)
T10	104 (26.6)	23 (5.9)	12 (3.1)	34 (8.7)	391 (100)
T11	79 (23.8)	20 (6.0)	13 (3.9)	31 (9.3)	332 (100)
T12	70 (25.5)	12 (4.4)	0 (0)	26 (9.5)	274 (100)
T13	58 (24.5)	9 (3.8)	4 (1.7)	20 (8.4)	237 (100)
T14	54 (28.3)	6 (3.1)	2 (1.0)	14 (7.3)	191 (100)
T15	41 (27.7)	6 (4.1)	1 (0.7)	17 (11.5)	148 (100)
T16	36 (30.0)	6 (5.0)	3 (2.5)	11 (9.2)	120 (100)
T17	26 (36.6)	2 (2.8)	2 (2.8)	7 (9.9)	71 (100)
T18	17 (32.1)	1 (1.9)	0 (0)	4 (7.5)	53 (100)
T19	9 (30.0)	1 (3.3)	0 (0)	1 (3.3)	30 (100)
T20	8 (30.8)	0 (0)	0 (0)	0 (0)	26 (100)
Total, n (%)	2650 (27.4)	593 (6.1)	473 (4.9)	929 (9.6)	9687 (100)

RIS: reference iron status; AID: absolute iron deficiency; FID: functional iron deficiency; HIS: high iron status; NC: not classified

Figure S1. Longitudinal data of Hs-CRP (A), serum iron (B), ferritin (C) and TSAT (D) during the follow-up. Data were presented as median±IQR.

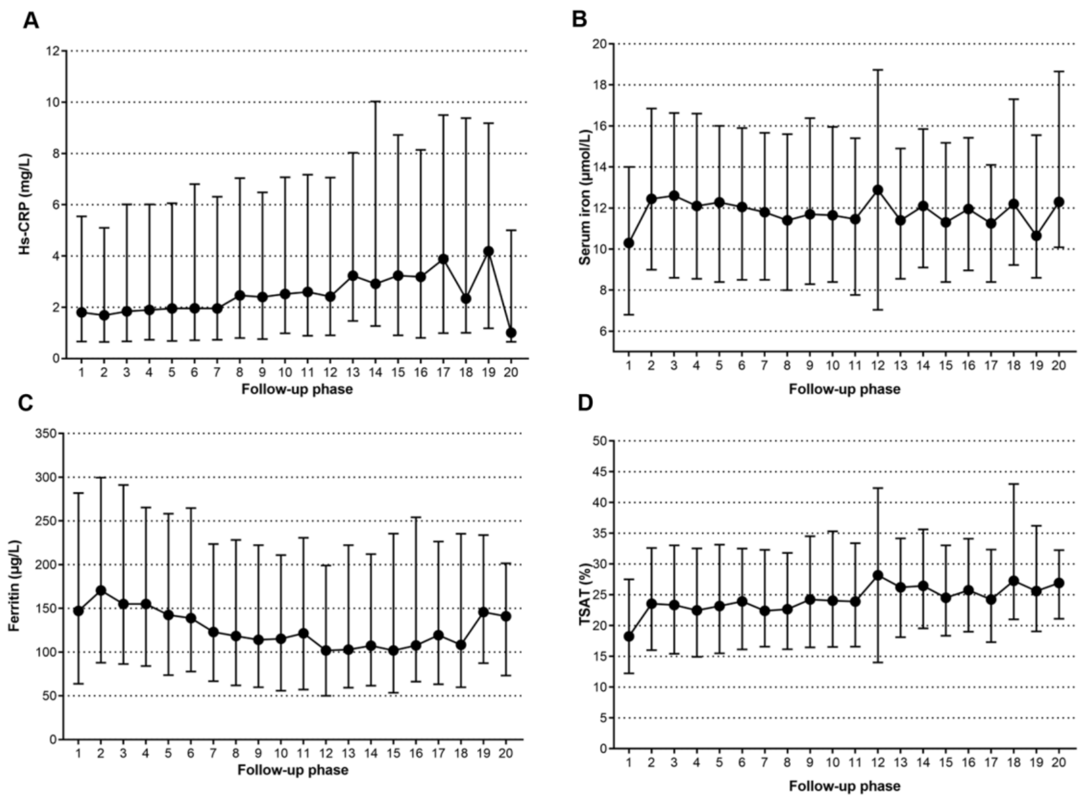

Supplement: Supplementary file 1 [file nutrients-14-01613-s001.zip › nutrients-1616670-supplementary.pdf]
